# Using administrative healthcare data to evaluate drug repurposing opportunities for cancer: the possibility of using beta-blockers to treat breast cancer

**DOI:** 10.3389/fphar.2023.1227330

**Published:** 2023-08-10

**Authors:** George S. Q. Tan, Edoardo Botteri, Stephen Wood, Erica K. Sloan, Jenni Ilomäki

**Affiliations:** ^1^ Centre for Medicine Use and Safety, Monash University, Parkville, VIC, Australia; ^2^ Section for Colorectal Cancer Screening, Cancer Registry of Norway, Oslo, Norway; ^3^ Research Department, Cancer Registry of Norway, Oslo, Norway; ^4^ Monash Institute of Pharmaceutical Sciences, Drug Discovery Biology Theme, Monash University, Parkville, VIC, Australia; ^5^ Division of Cancer Surgery, Peter MacCallum Cancer Centre, Melbourne, VIC, Australia

**Keywords:** drug repurposing, real-world data, administrative healthcare data, breast cancer, beta-blocker, cancer mortality, cancer survival

## Abstract

**Introduction:** Cancer registries and hospital electronic medical records are commonly used to investigate drug repurposing candidates for cancer. However, administrative data are often more accessible than data from cancer registries and medical records. Therefore, we evaluated if administrative data could be used to evaluate drug repurposing for cancer by conducting an example study on the association between beta-blocker use and breast cancer mortality.

**Methods:** A retrospective cohort study of women aged ≥50 years with incident breast cancer was conducted using a linked dataset with statewide hospital admission data and nationwide medication claims data. Women receiving beta blockers and first-line anti-hypertensives prior to and at diagnosis were compared. Breast cancer molecular subtypes and metastasis status were inferred by algorithms from commonly prescribed breast cancer antineoplastics and hospitalization diagnosis codes, respectively. Subdistribution hazard ratios (sHR) and corresponding 95% confidence intervals (CIs) for breast cancer mortality were estimated using Fine and Gray’s competing risk models adjusted for age, Charlson comorbidity index, congestive heart failure, myocardial infraction, molecular subtype, presence of metastasis at diagnosis, and breast cancer surgery.

**Results:** 2,758 women were hospitalized for incident breast cancer. 604 received beta-blockers and 1,387 received first-line antihypertensives. In total, 154 breast cancer deaths were identified over a median follow-up time of 2.7 years. We found no significant association between use of any beta-blocker and breast-cancer mortality (sHR 0.86, 95%CI 0.58–1.28), or when stratified by beta-blocker type (non-selective, sHR 0.42, 95%CI 0.14–1.25; selective, sHR 0.95, 95%CI 0.63–1.43). Results were not significant when stratified by molecular subtypes (e.g., triple negative breast cancer (TNBC), any beta blocker, sHR 0.16, 95%CI 0.02–1.51).

**Discussion:** It is possible to use administrative data to explore drug repurposing opportunities. Although non-significant, an indication of an association was found for the TNBC subtype, which aligns with previous studies using registry data. Future studies with larger sample size, longer follow-up are required to confirm the association, and linkage to clinical data sources are required to validate our methodologies.

## Introduction

Drug repurposing is a promising supplement to *de novo* drug discovery and development due to time and cost savings (Parvathaneni et al., 2019; Pushpakom et al., 2019; Roy et al., 2021). Repurposed drugs have lower attrition rates across clinical trial and regulatory approval phases compared to their *de novo* counterparts (Parvathaneni et al., 2019; Pushpakom et al., 2019; Roy et al., 2021). This is particularly relevant to oncology, where on average only 3.4% of drugs successfully traverse from Phase I clinical trials to market approval (Wong et al., 2019). Real-world data, including administrative healthcare data, may be used to discover or validate drug repurposing candidates via pharmaco-epidemiological analyses (Pushpakom et al., 2019; Ozery-Flato et al., 2020; Xu et al., 2020; Chen et al., 2021). The benefits of administrative healthcare data are longitudinal individual-level records, large population-based sample sizes, and widespread availability (Katkade et al., 2018; Ilomaki et al., 2020; Franklin et al., 2021; Tan et al., 2023). These advantages are attributable to embedded data collection processes during routine provision of healthcare.

Beta-blockers have been identified as potential repurposing candidates for cancer due to emerging evidence from preclinical studies (Sloan et al., 2010; Cole and Sood, 2012; Magnon et al., 2013; Creed et al., 2015; Chang et al., 2016; Le et al., 2016), retrospective observational studies across various cancer types (e.g., melanoma (De Giorgi et al., 2013), ovarian cancer (Watkins et al., 2015; Couttenier et al., 2019), colorectal cancer (Jansen et al., 2014), prostate cancer (Lu et al., 2015; Malik et al., 2023), and breast cancer (Botteri et al., 2013; Lofling et al., 2022)), and recent prospective clinical trials (Shaashua et al., 2017; Hiller et al., 2020). Despite advances in breast cancer therapeutics, metastasis remains the key prognostic marker for poor survival and the main cause of death (Weigelt et al., 2005; Jung et al., 2011). Preclinical evidence suggests that beta-blockade reduces metastasis by limiting tumour cell invasion, inflammation in the tumor microenvironment, and lymphatic vasculature remodelling (Palm et al., 2006; Sloan et al., 2010; Creed et al., 2015; Chang et al., 2016; Le et al., 2016). Additionally, beta-blockers may interact with breast cancer treatments to exert their protective effects (Pasquier et al., 2011; Reeder et al., 2015; Chang et al., 2023). However, a meta-analysis of epidemiological and perioperative studies by Yap et al. (2018) raised the possibility that the effects of beta-blockers on cancer outcomes may be tumor type-specific, as significant benefits were observed only in melanoma and ovarian cancer (Yap et al., 2018). Several retrospective observational studies found that beta-blocker use is associated with improved cancer outcomes in breast cancer (Powe et al., 2010; Barron et al., 2011; Melhem-Bertrandt et al., 2011; Botteri et al., 2013; Sorensen et al., 2013; Choy et al., 2016; Gillis et al., 2021; Lofling et al., 2022), while other studies have reported no significant association (Cardwell et al., 2013; Ganz et al., 2011; Sakellakis et al., 2014; Modi et al., 2020-; Cardwell et al., 2016). Moreover, meta-analyses by Løfling et al. (2022), Caparica et al. (2021) and Spini et al. (2019) have demonstrated more consistent protective effects of beta-blockers amongst women with triple negative breast cancer (TNBC) (Spini et al., 2019; Caparica et al., 2021; Lofling et al., 2022). Previous observational studies predominantly used data from population-based cancer registries and hospital electronic medical records (Mohammadzadeh et al., 2017), which contain detailed histopathological and other clinical information about cancer. However, cancer registry data may be unavailable or unfit for research purposes, especially in some developing countries (Valsecchi and Steliarova-Foucher, 2008; Abdul-Sater et al., 2021).

There is currently a paucity of studies using administrative healthcare data alone to investigate oncology-related repurposing hypotheses. To address this, we aimed to evaluate a drug repurposing opportunity in cancer using administrative healthcare data by analysing the association between beta-blocker use and breast cancer survival. We used an active comparator study design that compared beta-blocker users to users of first-line anti-hypertensives to reduce confounding and healthy user bias (Lund et al., 2015). In the absence of detailed cancer-related clinical information in the administrative dataset, we used longitudinal hospital admission data and prescription medication claims to infer incident cancer cases, baseline medication use and comorbidities, as well as breast cancer molecular subtypes.

## Materials and methods

### Data sources

We conducted a cohort study using hospitalization data from the Victorian Admitted Episodes Dataset (VAED), linked to medication claims data from the Australian Pharmaceutical Benefits Scheme (PBS) and death data from the National Death Index. Probabilistic linkage was performed by the Australian Institute of Health and Welfare (Madden et al., 2022), based on first name, middle names, surname, date of birth, date of death, street address, suburb, postcode, and sex. This linked dataset consists of 450,958 individuals discharged from any public or private hospital in the state of Victoria, Australia (total population of 6.5 million) between 1 July 2012 and 30 June 2018, and had one of the following four conditions recorded at the hospital: diabetes complication, acute coronary syndrome, stroke, or hip fracture.

The VAED data contains hospital admission data for each person from 1 July 2006 to 30 June 2018, including admission and discharge dates, principal and up to 39 additional diagnostic codes based on International Statistical Classification of Diseases and Related Health Problems, 10th Revision, Australian Modification (ICD-10-AM) (The International Statistical Classification, 2019), procedural codes based on Australian Classification of Health Interventions (ACHI) (Australian Classification, 2019), demographic information including age in 5-year groups and sex. The PBS is a nationwide single-payer system that subsidizes prescription medications for Australian citizens, permanent residents, and people from countries with reciprocal healthcare arrangements with Australia. Linkage to the PBS allowed identification of prescription medicine claims from 1 January 2005 to 30 June 2018, including information on the medication name, strength, quantity supplied, and dispensing date (Mellish et al., 2015). The National Death Index provided the date and causes of death. Cause of death includes the underlying cause and other causes of death.

### Study population

The study cohort was defined as women, aged ≥50 years discharged from any Victorian hospital between 1 November 2012 to 30 June 2018 with breast cancer as the principal diagnosis (ICD-10-AM code C50). This start date was necessary because medications costing less than the general co-payment amount were not recorded in the dataset for general beneficiaries prior to 1 July 2012 (Mellish et al., 2015), and we required a 120-day window for medication exposure ascertainment prior to hospital admission. The first hospitalization with breast cancer recorded as a principal diagnosis during the study period was defined as the index hospitalization. The discharge date of the index hospitalization was defined as the index date. The cohort was limited to women aged ≥50 years to exclude premenopausal women because of the use of different treatment strategies, poorer prognoses, and to reduce heterogeneity between beta-blocker users and comparators (Azim et al., 2016). People with prior hospital admissions with cancer recorded as any diagnoses since 1 July 2006 or dispensing records of any antineoplastics from 1 January 2005 until the index hospitalization were excluded to obtain an incident cancer cohort.

### Exposure

**FIGURE 1:**
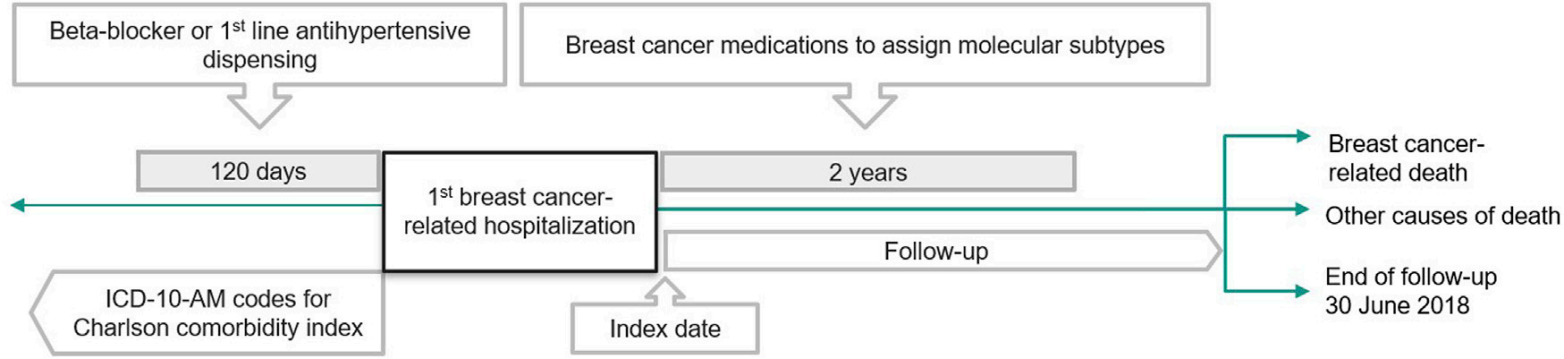
Study flowchart.

We determined exposure to beta-blockers and comparator medications (first-line antihypertensives), using a 120-day lookback period from the admission date (inclusive) of index hospitalization (Figure 1). Pre-diagnostic use of beta-blocker was chosen as the exposure of interest to address the hypothesis that beta-blockers have synergistic effects with breast cancer treatment during the diagnostic period (Pasquier et al., 2011; Reeder et al., 2015). Beta-blocker exposure was defined by having at least one beta-blocker dispensing record within the lookback period (Supplementary Appendix S1). We classified people as having received either selective or nonselective beta blockers based on the most recent dispensing date. To reduce confounding by indication, the comparators were defined as people with at least one dispensing record of a first-line anti-hypertensive medication with no beta-blocker dispensing record during the look-back period. Comparator medications were selected based on first-line recommendations in the Australian Therapeutic Guidelines (Blood Pressure Reduction, 2021), including angiotensin-converting enzyme inhibitors, angiotensin-receptor blockers, thiazide-like diuretics and dihydropyridine calcium channel blockers. All medications were defined based on the Anatomical Therapeutic Chemical (ATC) classification of medications (Supplementary Appendix S1 and S2).

### Covariates

Molecular subtypes of breast cancer diagnoses were inferred using an algorithm of breast cancer medications (Table 1), based on Australian clinical practice guidelines (eviQ Cancer Institute NSW, 2021; Immunomodulators and antineoplastics, 2021). This was completed using medication dispensing records from the 2 years during and after the index date, including medications that target the estrogen and/or progesterone receptor, namely, selective estrogen receptor modulators (tamoxifen, toremifene), aromatase inhibitors (letrozole, anastrozole, exemestane) and gonadotrophin-releasing hormone agonist (goserelin), and HER2 targeting monoclonal antibodies (trastuzumab and pertuzumab).

**TABLE 1:** Molecular subtype classification based on breast cancer medications.

Breast cancer molecular subtype	Estrogen and/or progesterone receptor targeting agent	HER2 targeting agent	Conventional chemotherapy^a^
Luminal (A/B)	+	+/−	+/−
HER2 positive	−	+	+/−
Triple negative	−	−	+

+, Presence of one or more dispensing record. −, Absence of a dispensing record. +/−, presence or absence of dispensing record.

^a^ Defined using ATC, codes starting with L01.

The Charlson comorbidity index by Quan et al. was used to identify 17 comorbidities based on ICD-10-AM codes from all hospitalizations on or before the index date (Supplementary Appendix S3) (Charlson et al., 1987; Quan et al., 2005). Weighted Charlson comorbidity scores were calculated, excluding cancer and metastasis because everyone in the cohort had one or both of these. Baseline metastasis was identified from the index hospitalization using ICD-10-AM codes C77 for regional lymph node metastasis, and C78, C79, and C80 for distant metastasis. Breast cancer surgery during index hospitalization was ascertained using recorded procedural codes. Breast cancer surgery codes include those for excision of lesions and mastectomy (Supplementary Appendix S4) (Victorian Cancer Quality, 2018).

### Outcomes

The primary outcome in this study was breast cancer mortality and the secondary outcome was all-cause mortality. Follow-up started from the index date until the end of the study period 30 June 2018. People who died prior to hospital discharge were excluded. Breast cancer death was determined using ICD-10-AM code C50 recorded in the National Death Index using both underlying and other recorded causes of death. We included other recorded causes of death in the definition to improve sensitivity as it has been reported that only 77.5% of breast cancer-related deaths had breast cancer coded as the “underlying” cause of death in the National Death Index (Multiple causes of death, 2012).

### Statistical analyses

Descriptive analyses were used to describe characteristics of the study cohort. Categorical variables are reported with frequencies and percentages; continuous variables with medians and interquartile ranges (IQR). Statistical significance in the cohort characteristics between the groups was determined using parametric or non-parametric tests as appropriate, with an alpha level of 0.05.

Multivariable analyses were conducted for all beta-blockers, and selective and nonselective beta-blockers separately. All models were adjusted for age group, weighted Charlson comorbidity index, congestive heart failure, myocardial infarction, baseline metastasis (no metastasis, regional or distant metastasis), breast cancer molecular subtypes and breast cancer surgery during the index hospitalization. Congestive heart failure and myocardial infarction were used as separate covariates due to both being the main indications for beta-blocker use. We carried out competing-risk analyses using Fine and Gray’s subdistribution hazards model to estimate the subdistribution hazards ratios (sHR) and 95% confidence intervals (CI) for breast cancer mortality (Fine and Gray, 1999). Deaths due to other causes were used as competing events in breast cancer mortality analyses. Cox proportional hazards model was used to estimate the hazard ratios (HR) and 95% CI for overall survival.

Stratified analyses were conducted for age groups (<65 years vs. ≥ 65 years), weighted Charlson comorbidity index (<3 and ≥3), hormone-receptor positive breast cancer, TNBC, and cancer baseline metastasis (no metastasis, regional or distant metastasis) to examine potential effect modification.

All analyses were conducted using SAS version 9.4 (SAS Institute Inc., Cary, NC, United States).

## Results

### Cohort description

**FIGURE 2:**
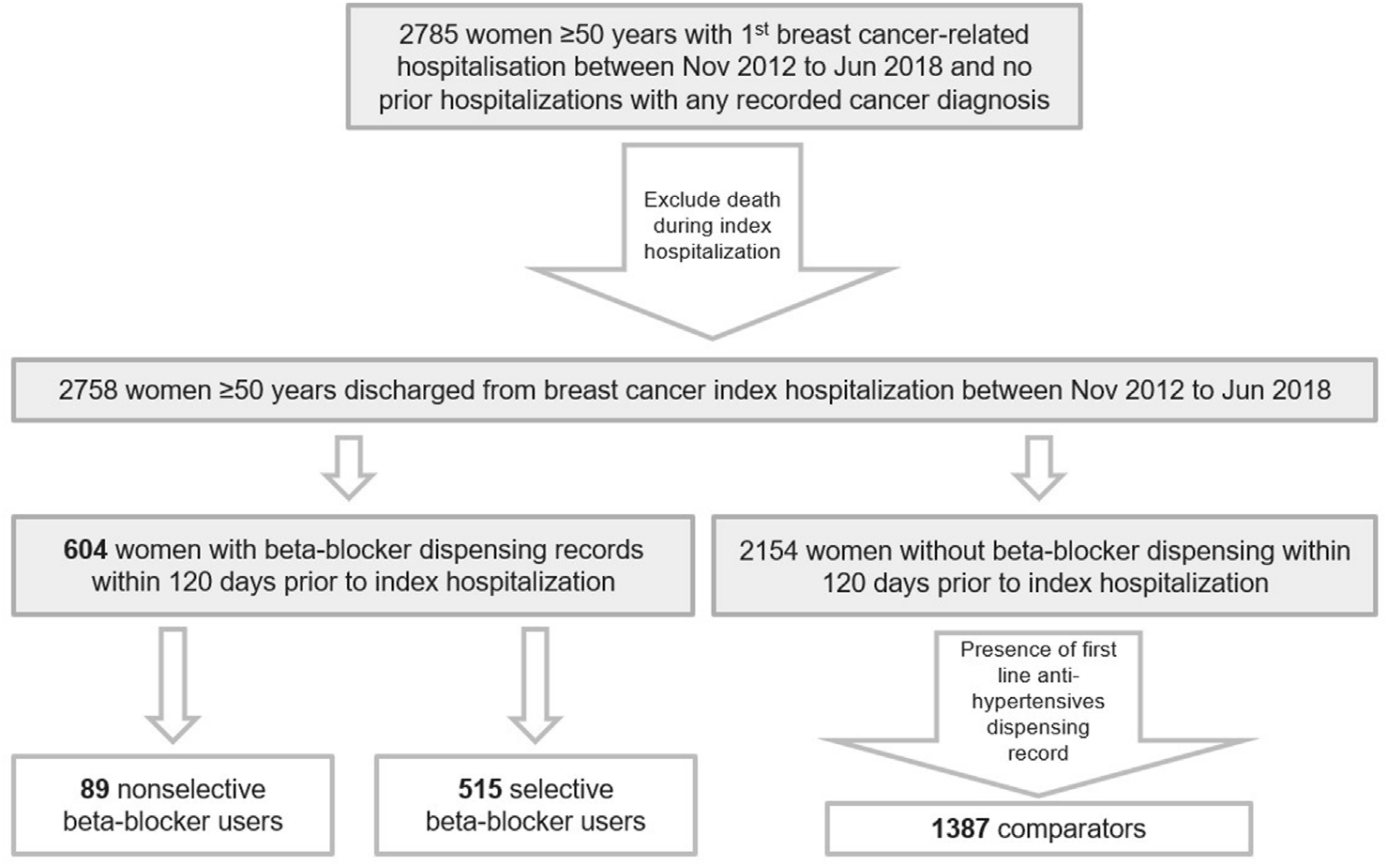
Cohort extraction process.

From a cohort of 2,758 women ≥50 years with breast cancer, there was a total of 604 beta-blocker users in our study cohort, including 89 nonselective and 515 selective beta-blocker users (Figure 2; Table 2). There were in total 1,387 users of first-line antihypertensives (active comparators). The median age of our study population was 71 years.

**TABLE 2:** Baseline characteristics of the study sample.

Characteristic	All beta-blocker users	Nonselective beta-blocker users	Selective beta-blocker users	Comparators	*p*-value
Total women, n	604	89	515	1387	—
Age group in years, n (%)					
50–69	204 (33.8)	27 (30.3)	177 (34.4)	467 (47.0)	<0.001^b^
70–79	227 (37.6)	34 (38.2)	193 (37.5)	480 (34.6)	0.2^b^
≥80	173 (28.6)	28 (31.5)	145 (28.2)	255 (18.4)	<0.001^b^
Weighted Charlson Comorbidity index^c^					
Median (IQR)	2.0 (2.0)	2.0 (2.0)	3.0 (4.0)	1.0 (2.0)	0.02^d^
0–1, n (%)	274 (45.4)	43 (48.3)	136 (26.41)	756 (54.5)	<0.001^b^
2–3, n (%)	193 (32.0)	27 (30.3)	187 (36.3)	443 (31.9)	0.99^b^
≥4, n (%)	137 (22.7)	19 (21.4)	192 (37.3)	188 (13.6)	<0.001^b^
Myocardial infarction, n (%)	72 (11.9)	9 (10.1)	63 (12.2)	56 (4.0)	<0.001^b^
Congestive heart failure, n (%)	93 (15.4)	11 (12.4)	62 (12.0)	68 (4.9)	<0.001^b^
Regional metastasis, n (%)	187 (31.0)	25 (28.1)	165 (32.0)	422 (30.4)	0.81^b^
Distant metastasis, n (%)	24 (4.0)	<6 (<6.7)^a^	<24 (<4.7)^a^	69 (5.0)	0.33^b^
Luminal breast cancer, n (%)	444 (73.5)	63 (70.8)	381 (74.0)	1059 (76.4)	0.18^b^
HER2 positive breast cancer, n (%)	15 (2.5)	<6 (<6.7)^a^	<15 (<2.9)^a^	35 (2.5)	0.96^b^
TNBC, n (%)	42 (7.0)	7 (7.9)	35 (6.8)	119 (8.6)	0.22^b^
Breast cancer surgery during index admission, n (%)	559 (92.6)	80 (89.9)	479 (93.0)	1273 (91.8)	0.56^b^

TNBC, triple negative breast cancer.

^a^ Small cell suppression (<6) required by Australian Institute of Health and Welfare.

^b^ Chi-square test.

^c^ Cancer and metastasis were excluded in the weighted Charlson comorbidity index.

^d^ Kruskall-Wallis test.

Beta-blocker users were older than the comparators (≥80 years old: 29% vs. 18%, *p* < 0.001). (Table 2). Beta-blocker users tended to have more comorbidities compared to comparators (weighted Charlson comorbidity index of ≥4: 23% vs. 14%, *p* = 0.005). Beta-blockers users were more likely to have congestive heart failure and a history of myocardial infarction than comparators (15% vs. 5%, *p* < 0.001; 12% vs. 4%, *p* < 0.001). Beta-blocker users and comparators had similar distributions of baseline metastasis, breast cancer molecular subtypes, and breast cancer surgery during the index hospitalization period.

### Breast cancer survival

There were in total 154 breast cancer deaths over the median follow-up time of 2.7 years (IQR 2.8 years, Table 3). After adjusting for all covariates, there was no difference in breast cancer survival between any beta-blocker users and the comparators (sHR 0.86, 95% CI 0.58–1.28). When stratified by beta-blocker type, there was no difference in breast cancer mortality between nonselective (sHR 0.42, 95% CI 0.14–1.25) or selective beta-blocker users (sHR 0.95, 95% CI 0.63–1.43) compared to the comparators.

**TABLE 3:** Crude mortality rate and hazard ratios by beta-blocker use.

	All beta-blockers users	Non-selective beta-blockers users	Selective beta-blocker users	Comparators
Total, n	604	89	515	1,387
Follow-up time (years), median (IQR)	2.72 (2.62)	2.57 (2.92)	2.77 (2.54)	2.73 (2.90)
Breast cancer survival				
Breast cancer-related deaths, n (%)	49 (8.1)	<6 (<6.7)^a^	<49 (<9.5)^a^	105 (7.6)
Unadjusted sHR (95% CI)	1.03 (0.74–1.45)	0.60 (0.22–1.63)	1.10 (0.78–1.56)	reference
Adjusted sHR^b^ (95% CI)	0.86 (0.58–1.28)	0.42 (0.14–1.25)	0.95 (0.63–1.43)	reference
All-cause survival				
All-cause deaths, n (%)	98 (16.2)	13 (14.6)	85 (16.5)	167 (12.0)
Unadjusted hazard ratios (95% CI)	1.33 (1.03–1.70)	1.28 (0.73–2.26)	1.33 (1.03–1.73)	reference
Adjusted hazard ratios^b^ (95% CI)	1.02 (0.79–1.32)	0.80 (0.45–1.42)	1.07 (0.81–1.40)	reference

CI, confidence interval; sHR, subdistribution hazard ratio; IQR, interquartile range.

^a^ Small cell suppression (<6) required by Australian Institute of Health and Welfare.

^b^ Adjusted for age group, weighted Charlson comorbidity index, congestive heart failure, myocardial infraction, metastasis at baseline (no metastasis vs. localized or distant metastasis), breast cancer molecular subtype and breast cancer surgery during the index hospitalization.

**FIGURE 3:**
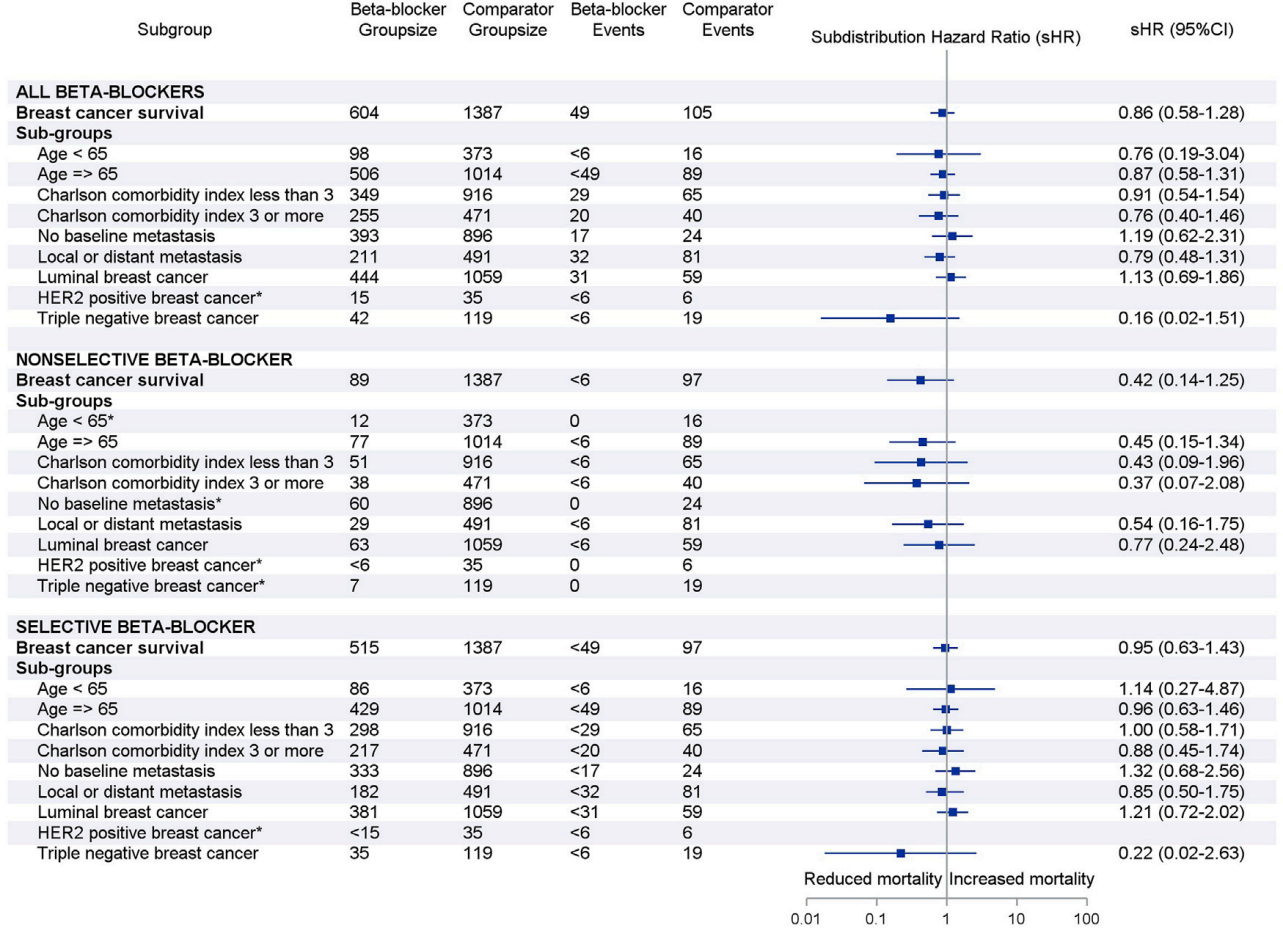
Forest plots of subgroup analyses by beta-blocker use. Adjusted subdistribution hazard ratios reported based on Fine and Gray’s subdistribution hazard model. Note: small cell suppression (<6) is required by Australian Institute of Health and Welfare. *Hazards ratio could not be estimated due to small number or lack of breast cancer deaths in subgroups.

Results were similar when stratified by age group, weighted Charlson comorbidity index, baseline metastasis and breast cancer molecular subtype (Figure 3). In particular, we did not find a significant association between beta blocker use and breast cancer survival in women with TNBC (any beta-blocker, sHR 0.16, 95% CI 0.02–1.51; selective beta-blocker, sHR 0.22, 95% CI 0.02–2.63). Results could not be determined for all HER2 positive subgroups and TNBC subgroup for non-selective beta-blocker users due to small number or lack of events.

### All-cause survival

There were 265 all-cause deaths over the same follow-up period (Table 3). In the multivariable analyses, there was no significant difference in all-cause mortality between any beta-blocker use (HR 1.02, 95% CI 0.79–1.32) compared to the comparators. Results remained similar when stratified by beta-blocker type: nonselective beta-blocker (HR 0.80, 95% CI 0.45–1.42) and selective beta-blocker (HR 1.07, 95% CI 0.81–1.40).

## Discussion

In this example using administrative healthcare data to investigate a drug repurposing candidate for cancer, we found no association between either use of any beta-blocker, nonselective beta-blocker use, or selective beta-blocker use, and short-term breast cancer mortality (median 2.7 years of follow-up). The results are aligned with a meta-analysis of 13 studies by Caparica et al. (2021) who reported no significant difference between beta blocker use and breast cancer mortality (Caparica et al., 2021). However, notably recent studies using cancer registry data or hospital electronic medical records with longer follow-up times and larger sample sizes have demonstrated that beta-blockers prolong breast cancer survival in TNBC patients. A recent meta-analysis of four independent studies by Løfling et al. (2022) reported a 26% reduced risk of breast cancer death in women with TNBC with the use of beta blockers compared to no use (Lofling et al., 2022). For example, Botteri et al. (2013) observed a 48% reduced risk of breast cancer death in women with TNBC who used any beta-blockers (n = 74) over 5 years (Botteri et al., 2013). Løfling et al. (2022) demonstrated a 34% improved breast cancer-survival with beta blocker use in women with TNBC (*n* = 312) over 5 years (Lofling et al., 2022). Our analyses indicated a possible reduction in breast cancer mortality for women with TNBC using beta-blockers compared to comparators (sHR 0.16, 95% CI 0.02–1.51), although results were not statistically significant. This may be due to short follow-up, small sample size, fundamental differences in study cohorts, and residual confounding (see limitations below).

The key advantage of administrative healthcare data over cancer registry data or electronic medical records is the presence of longitudinal and comprehensive records of medication use. In this study, we ascertained baseline use of beta-blockers by using longitudinal prescription medication claims. This allowed us to accurately identify the type of beta-blocker use (selective vs. non-selective) dispensed prior to breast cancer diagnosis. Studies using cancer registry data without linkage to medication claims often rely on patient self-reports or hospital electronic medical records to identify baseline use of medications (Pop et al., 2019). Self-reporting in cancer registry data is subject to recall bias. Moreover, hospital electronic medical records may not have exact information when the medication was initiated and may include initiation of exposure medication use after a breast cancer diagnosis. Ascertaining exposure of medications after the index date may lead to an immortal time bias, where people by design need to survive event-free until the exposure starts at a time after the index date (Suissa, 2008). Immortal time bias could distort results towards favourable drug-outcome associations (Weberpals et al., 2016). This was demonstrated in a meta-analysis by Zhong et al. (2016), which found that post- but not pre-diagnostic use of beta-blockers was associated with improved cancer survival (Zhong et al., 2016).

Cancer registries remain the gold standard real-world data source for evaluating drug repurposing opportunities for cancer due to rich clinical information on cancer diagnosis and management. However, administrative healthcare data are considered as alternative data sources if cancer registry data are unavailable or unfit for research purposes, especially in developing countries (Valsecchi and Steliarova-Foucher, 2008; Abdul-Sater et al., 2021). Several previous studies have used administrative data to evaluate drug repurposing opportunities for cancers other than breast cancer (Chen et al., 2019; Chen et al., 2020; Ng et al., 2021; Park et al., 2021). Some of these studies even have enriched administrative healthcare data (Ng et al., 2021), or linkage to external registry to provide or confirm cancer-related clinical information (Chen et al., 2019; Chen et al., 2020).

Due to the lack of cancer-specific histopathological clinical data in our dataset, we acquired information on incident breast cancer cases from longitudinal hospitalization data and prescription medication claims. We identified the first hospitalization record where breast cancer was recorded as a primary diagnosis and used this as a proxy for the date of breast cancer diagnosis. Cancer registries usually record incident cancer cases and provide accurate dates of diagnosis (Yuen et al., 2011; Okuyama et al., 2018). However, Yuen et al. have demonstrated good sensitivity (84.8%) and specificity (99.9%) between administrative data and actual recorded dates of diagnosis in cancer registry (Yuen et al., 2011).

We used diagnosis and procedural codes from hospitalization data and prescription medication claims data to infer breast cancer molecular subtypes and staging. We developed a novel algorithm to estimate breast cancer molecular subtypes from commonly prescribed antineoplastics used to treat each molecular subtype of breast cancer. Molecular subtype was used to stratify and adjust our analyses. Moreover, we used the presence of breast cancer surgery at index hospital admission and diagnosis codes for metastasis to infer early breast cancer cases. The meta-analysis by Caparica et al. (2021) evaluated the association between beta-blocker use and breast cancer survival in early breast cancer cases given their better prognoses (Caparica et al., 2021). Given that early breast cancer cases were likely to have localized operable tumors or no metastasis, we included both the presence of breast cancer surgery during index hospitalization and metastasis as independent covariates for adjustment in our analyses.

Strengths of our study include the use of active comparators and competing risk analyses. The use of active comparators minimizes confounding by indication (Lund et al., 2015), and other differences in baseline characteristics between the exposure groups. The active comparators also minimize healthy user bias as all women in the study were active users of preventive cardiovascular medications and the healthcare system (Lund et al., 2015). Despite this, a higher proportion of beta-blocker users had congestive heart failure and higher weighted Charlson comorbidity scores than the comparators, therefore, our model adjusted for both of these. We also considered competing risks in our time-to-event survival analyses using Fine and Gray’s subdistribution hazard model. Competing risk events can lead to overestimation of risk when using the conventional Cox proportional hazards regression model (Tan et al., 2018). This is especially true when analyzing a cohort with a substantial proportion of older people, as the incidence of diseases that are attributable to aging and frailty increases (Tan et al., 2018).

There are important limitations to this study. First, the limited sample size and short follow-up time (median 2.7 years) led to underpowered analyses. Second, the study cohort included people admitted to hospitals with diabetes, acute coronary syndrome, stroke, or hip fracture only. This limited the ability to generalize the results to a general population of women with breast cancer. To address these two limitations, future studies would benefit from using larger scale administrative data from a nationwide or general population-based cohort to both increase the power of study and generalizability of results. Third, there is the potential of confounding by severity as beta-blockers are not first-line anti-hypertensives and are prescribed for people with additional cardiovascular comorbidities, notably ischaemic heart disease and congestive heart failure (Blood Pressure Reduction, 2021). We considered first-line anti-hypertensives as the best possible active comparator medications as they have previously been used in other studies to reduce confounding (Gillis et al., 2021; Lofling et al., 2022), and included myocardial infarction and congestive heart failure as covariates in the models. Furthermore, there could be residual confounding due to undetermined histopathological characteristics of breast cancer tumors, for example, tumor size and Ki-67 expression, which are conventionally available from cancer registry data but not in our dataset. Fourth, we used dispensing records during follow-up to estimate breast cancer molecular subtype, which may lead to selection bias in the stratified analyses by molecular subtypes. This is because people need to survive during follow-up until receiving the breast cancer treatments (Acton et al., 2023). Furthermore, we were not able to validate the sensitivity and specificity of the algorithm used in inferring breast cancer molecular subtypes as we did not have information on the subtypes available. Future validation studies with data linkage to cancer registries are required to confirm the validity of the algorithm. Moreover, we were unable to classify breast cancer molecular subtype for 14% of women as they did not have dispensing records of either breast cancer antineoplastics or conventional chemotherapy. Therefore, the algorithm could not infer the breast cancer molecular subtypes for women who have undergone surgery or radiotherapy only. Lastly, given that this study only includes data from patients treated until mid-2018, our results do not include the effects of emerging immunotherapies used since 2019 (Barzaman et al., 2021). Preclinical studies have reported that beta-blockade enhances the response to immunotherapy, for example, anti-CTLA4 therapy (Fjaestad et al., 2022), raising the possibility that beta blocker use might enhance new immunotherapy regimens.

## Conclusion

Use of administrative data as an alternative to registry data increases opportunities to identify novel drug repurposing candidates. In addition to being more widespread and accessible than registry data, administrative data often has longitudinal and comprehensive records of medication use that can be used to ascertain medication exposure. The current study emphasizes the need for use of large administrative datasets to provide sufficient power to identify associations. The current study describes the development of methodologies to infer cancer-related clinical information from variables available in administrative dataset. Future studies with linkage to clinical data sources are required to validate these methodologies.

## Data Availability

The data analyzed in this study is subject to the following licenses/restrictions; The data that support the findings of this study are available from the Australian Institute of Health and Welfare. Restrictions apply to the availability of these data, which were used under license for this study. Data are available from the authors only with the permission of the Australian Institute of Health and Welfare. Requests to access these datasets should be directed to the Australian Institute of Health and Welfare.
