# Supplementary material for: Using administrative healthcare data to evaluate drug repurposing opportunities for cancer: the possibility of using beta-blockers to treat breast cancer
Source: Front Pharmacol. 2023 Aug 10;14:1227330. doi: 10.3389/fphar.2023.1227330 (PMC10448902; doi:10.3389/fphar.2023.1227330)
Supplement: Supplementary file 1 [file DataSheet1.docx]

# Supplementary materials

## Appendix 1

Table: Beta blockers

| Beta blocker type | Drug Name | ATC code |
| --- | --- | --- |
| Selective | Atenolol | C07AB03 |
| Selective | Metoprolol tartrate | C07AB02 |
| Selective | Bisoprolol | C07AB07 |
| Selective | Metoprolol succinate | C07AB02 |
| Selective | Nebivolol | C07AB12 |
| Nonselective | Labetalol | C07AG01 |
| Nonselective | Sotalol | C01BD, C07AA07 |
| Nonselective | Propranolol | C07AA05 |
| Nonselective | Carvedilol | C07AG02 |

## Appendix 2

Table: First-line antihypertensives used as active comparators

| Drug name | ATC code |
| --- | --- |
| Amlodipine | C08CA01 |
| Amlodipine + atorvastatin | C10BX03 |
| Amlodipine + valsartan | C09DB01 |
| Amlodipine + valsartan + hydrochlorothiazide | C09DX01 |
| Candesartan | C09CA06 |
| Candesartan + hydrochlorothiazide | C09DA06 |
| Captopril | C09AA01 |
| Chlortalidone | C03BA04 |
| Cilazapril monohydrate | C09AA08 |
| Enalapril | C09AA02 |
| Enalapril + hydrochlorothiazide | C09BA02 |
| Eprosartan | C09CA02 |
| Eprosartan + hydrochlorothiazide | C09DA02 |
| Felodipine | C08CA02 |
| Fosinopril | C09AA09 |
| Fosinopril + hydrochlorothiazide | C09BA09 |
| Hydrochlorothiazide | C03AA03 |
| Indapamide | C03BA11 |
| Irbesartan | C09CA04 |
| Irbesartan + hydrochlorothiazide | C09DA04 |
| Lercanidipine | C08CA13 |
| Lercanidipine + enalapril | C09BB02 |
| Lisinopril | C09AA03 |
| Losartan | C09CA01 |
| Nifedipine | C08CA05 |
| Olmesartan | C09CA08 |
| Olmesartan + amlodipine + hydrochlorothiazide | C09DX03 |
| Olmesartan medoxomil + amlodipine | C09DB02 |
| Olmesartan medoxomil + hydrochlorothiazide | C09DA08 |
| Perindopril | C09AA04 |
| Perindopril + amlodipine | C09BB04 |
| Perindopril + indapamide | C09BA04 |
| Quinapril | C09AA06 |
| Quinapril + hydrochlorothiazide | C09BA06 |
| Ramipril | C09AA05 |
| Ramipril + felodipine | C09BB05 |
| Telmisartan | C09CA07 |
| Telmisartan + amlodipine | C09DB04 |
| Telmisartan + hydrochlorothiazide | C09DA07 |
| Trandolapril | C09AA10 |
| Trandolapril + verapamil | C09BB10 |
| Valsartan | C09CA03 |
| Valsartan + hydrochlorothiazide | C09DA03 |
| Valsartan + sacubitril | C09DX04 |

## Appendix 3

Table: Charlson comorbidity index

| Charlson comorbidity | Charlson weight |
| --- | --- |
| Myocardial infarction | 1 |
| Congestive heart failure | 1 |
| Peripheral vascular disease | 1 |
| Cerebrovascular disease | 1 |
| Dementia | 1 |
| Chronic pulmonary disease | 1 |
| Rheumatologic disease | 1 |
| Peptic ulcer disease | 1 |
| Mild liver disease | 1 |
| Diabetes without chronic complications | 1 |
| Diabetes with chronic complications | 2 |
| Hemiplegia or paraplegia | 2 |
| Renal disease | 2 |
| Any malignancy, including leukemia and lymphoma ^a^ | 2 |
| Moderate or severe liver disease | 3 |
| Metastatic solid tumor ^a^ | 6 |
| AIDS/HIV | 6 |

^a^ Cancer and metastases were excluded in the weighted Charlson comorbidity index

Source: Charlson ME, Pompei P, Ales KL, MacKenzie CR. A new method of classifying prognostic comorbidity in longitudinal studies: development and validation. *J Chronic Dis.* 1987;40(5):373-383.

## Appendix 4

Table: Australian Classification of Health Intervention (ACHI) codes for breast cancer surgery.

| Description | ACHI Code |
| --- | --- |
| Complete excision of lesion with guidewire | 3150000 |
| Re-excision of lesion of breast | 3151500 |
| Total mastectomy (unilateral) | 3151800 |
| Total mastectomy (bilateral) | 3151801 |
| Subcutaneous mastectomy (unilateral) | 3152400 |
| Subcutaneous mastectomy (bilateral) | 3152401 |
| Complete excision of lesion without guidewire | 3153600 |
